# Iatrogenic Right-Sided Pneumothorax Presenting as ST-Segment Elevation: A Rare Case Report and Review of Literature

**DOI:** 10.1155/2017/3291751

**Published:** 2017-03-26

**Authors:** Bashar Alzghoul, Ayoub Innabi, Anusha Shanbhag, Kshitij Chatterjee, Farah Amer, Nikihil Meena

**Affiliations:** ^1^Department of Internal Medicine, University of Arkansas for Medical Sciences (UAMS), Little Rock, AR, USA; ^2^Faculty of Medicine, University of Jordan, Amman, Jordan; ^3^Division of Pulmonary and Critical Care Medicine, University of Arkansas for Medical Sciences (UAMS), Little Rock, AR, USA

## Abstract

Pneumothorax is a well-recognized complication of central venous line insertion (CVL). Rarely, pneumothorax can lead to electrocardiogram (ECG) findings mimicking ST-segment elevation myocardial infarction. We present a 63-year-old man with iatrogenic right-sided pneumothorax who developed ST-segment elevation on a 12-lead ECG suggestive of myocardial infarction. The ECG findings completely resolved after needle decompression and chest tube placement. This case points up this rare electrocardiographic finding with discussion of possible mechanisms and differential diagnosis.

## 1. Introduction

ST-segment elevation when seen on telemetry and confirmed on a 12-lead electrocardiogram (ECG) should alarm the physicians about the possibility of acute myocardial infarction, which warrants rapid intervention. Rarely, other etiologies may lead to rapid onset ST-segment elevation [1]. We present a rare case of iatrogenic pneumothorax with ST-elevation that completely resolved after decompression.

## 2. Case Presentation

**Figure 1:**
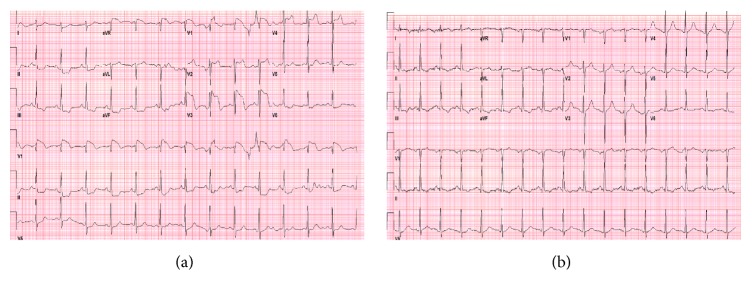
(a) Electrocardiogram showing ST-segment elevation in anterior leads with reciprocal changes in inferior leads (b) electrocardiogram after needle decompression and chest tube placement showing complete resolution of ST-segment elevation in anterior leads.

**Figure 2:**
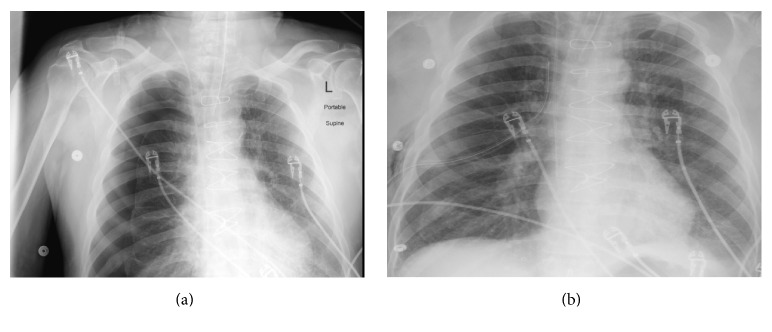
(a) Chest X-Ray showing large right-sided pneumothorax with lung margin collapse more than 4 cm from the chest wall uniformly. (b) Chest X-Ray showing Interval placement of chest tube with tip in the right suprahilar area with near-complete lung reexpansion.

A 63-year-old male with history of severe COPD, hypertension, and coronary artery disease presented with worsening dyspnea of one-week duration and vomiting and fever for two days. In the emergency department, his vital signs were stable except for temperature of 100.6 f (38.1 c). Oxygen saturation was 96% on 4L Nasal Cannula (NC). Physical examination revealed crackles heard over left lower hemithorax. Laboratory studies showed an arterial blood gas (ABG) of 7.39/49/48/29 on 4 NC, white blood cells count of 12.3 K/*μ*L, hemoglobin level of 15.3 g/dL, and troponin level of 0.04 ng/mL. Electrolytes panel showed no abnormality. Chest radiograph showed left lower lobe pneumonia with small pleural effusion. Angiogram Computed Tomography (CT) of the chest was negative for pulmonary embolism. Intravenous (IV) antibiotics were started. Baseline ECG showed sinus tachycardia with old T-wave inversions in inferolateral leads. Few hours later, patient developed hypotension refractory to IV fluids, necessitating placement of a central venous line (CVL). Soon after placing a right internal jugular CVL, the patient developed hypoxemic respiratory failure and was intubated immediately. Physical examination revealed blood pressure of 95/49 mmHg and heart rate of 78 beats per minute (bpm) with decreased breath sounds over the right anterior chest along with hyperresonance to percussion. Telemetry monitor showed ST-segment elevation. A 12-lead ECG was obtained (Figure 1(a)) showing ST-segment elevation in anterior leads with reciprocal T-wave changes in inferior leads. Chest radiograph showed large right-sided pneumothorax (Figure 2(a)). Needle decompression followed by chest tube placement (Figure 2(b)) was done immediately with rapid clinical improvement and resolution of ECG changes (Figure 1(b)). Repeat vital signs after chest tube placement showed blood pressure of 99/53 mmHg with heart rate of 102 bpm. Troponin level remained within the normal range.

## 3. Discussion

Central venous line (CVL) insertion is a common procedure in the intensive care unit setting. Pneumothorax is a well-recognized complication of CVL insertion [2, 3]. Previous reports described ECG changes in pneumothorax, and there are only scarce reports that have described ECG changes in iatrogenic pneumothorax [4, 5].

ECG changes are seen in approximately 25% of the Pneumothorax cases [6]. ECG findings range from right axis deviation, QRS alterations, and T-wave inversions to ST-segment changes including ST-segment elevation, which can imitate acute myocardial infarction [1, 4, 7]. ECG findings mimicking ST-segment elevation myocardial infarction have been reported in rare cases [1, 8, 9]. On reviewing 40 patients with spontaneous pneumothorax—both right- and left-sided—Krenke and colleagues found only 1 patient with T-wave inversion and no patients with ST-segment elevation [6]. Among the reported cases, most patients had preexisting coronary artery disease and COPD. However, a case reported by Shiyovich and colleagues did report ST-segment elevations due to pneumothorax in a young 23-year-old relatively healthy man with no past medical problems [1]. Numerous theories have been deduced to explain the associated ECG findings. The cause of brief ECG changes has been mainly attributed to build-up of intrapleural air, which can shift the cardiac silhouette and exert pressure on the heart and coronary vessels precipitating ischemia. Hypotension and decreased venous return caused pathologically by pneumothorax contribute to decreased cardiac output and tachycardia further adding to ischemia due to elevated myocardial oxygen demand [1]. Our patient was hypotensive secondary to sepsis before the CVL was placed and there was no significant change in blood pressure or heart rate noticed with the development of pneumothorax. In literature, ECG findings associated with right-sided pneumothorax not only have been reported less but also have been noted to be less pronounced when compared to a left-sided pneumothorax [8]. Uniquely, our patient showed significant ST-segment elevations in presence of right-sided pneumothorax and these changes reversed with resolution of pneumothorax.

ST-segment elevation has been reported in diverse pathologies including hyperkalemia, early repolarization, left bundle branch block, acute pericarditis, intracerebral bleeding, and Brugada syndrome [1, 10]. The importance of early revascularization in ST-segment elevation myocardial infarction mandates keeping it high on a clinician's differential diagnosis. However, one should be aware of other causes of ST-segment elevation to avoid delay in management. In our patient, pneumothorax was promptly decompressed but delay in recognizing such condition could be life threatening.

## 4. Conclusion

Practitioners should be aware about pneumothorax as an immediate complication of central venous line insertion and that it could present as ST-segment elevation so that a delay in diagnosis and treatment can be prevented. The whole clinical picture including patient's history and review of laboratory and radiological investigations is essential. ST-segment elevation should be scrutinized in relation to the clinical presentation and not as a diagnostic indicator of myocardial infarction on its own.
